# Exposure to e-cigarette content on social media and its association with risk perception, perceived benefits, and vaping perceptions among college students: a cross-sectional study

**DOI:** 10.3389/fpubh.2026.1815287

**Published:** 2026-06-04

**Authors:** Saud A. Alsulaiman, Ezaddeen S. Almutairi

**Affiliations:** Department of Mass Communication, College of Humanities and Social Sciences, Riyadh, King Saud University, Riyadh, Saudi Arabia

**Keywords:** college students, e-cigarette, perceived benefits, risk perceptions, social media use, vaping

## Abstract

**Introduction:**

This study investigates college students' overall perceptions of vaping, risk perceptions, and perceived benefits of e-cigarettes through the lens of the Health Belief Model (HBM), in relation to social media use and exposure to e-cigarette-related conten.

**Methods:**

This cross-sectional study used validated and reliable measures to assess perceptions of e-cigarettes among college students (*n* = 1,897) at a large university in Saudi Arabia via an online survey. Descriptive and inferential statistical analyses were performed, including bivariate and multivariate analyses, such as exploratory factor analysis, Spearman's rank correlation coefficient, Mann-Whitney U tests, linear regression, and binary logistic regression.

**Results:**

The findings suggested that the majority of college students (90%) use social media daily, and more than 57% of students who had seen e-cigarette-related content reported encountering it through influencer or followed accounts. The findings showed that exposure to e-cigarette content on social media was not associated with regular vaping, whereas with modest explanatory power, greater risk perception was associated with lower vaping use among college students [OR = 0.93, 95% CI (0.88, 0.97), *p* = .002]. Most college students (83%) had never vaped or smoked, yet those who vape had higher perceived benefits and lower risk perceptions than those who had not vaped.

**Conclusion:**

Unlike other studies conducted among teens and adolescents, our study did not find significant relationships between exposure to e-cigarette content on social media and vaping use. However, perceived benefits and risk perceptions were found to be significantly associated with the overall vaping perceptions and vaping use among college students. The study ultimately shows that although exposure to e-cigarette content is common on social media, it does not appear to meaningfully shape how college students judge the risks of vaping.

## Introduction

The use of electronic cigarettes (e-cigarettes), commonly known as vaping, has become popular among young people worldwide. More than 88 countries have no minimum age requirement for purchasing and consuming these harmful products (1). This has resulted in widespread use of massive advertising campaigns promoting tobacco products on social media. It is estimated that influencers promote more than 16,000 different flavors of e-cigarettes to target youth and children due to the large number of consumers (1). In 2021, it is estimated that there were more than 82 million people worldwide using vaping, and the number is expected to rise (2). In the US, for instance, more than 2 million middle and high school students use vaping (3, 4). The literature suggests that the use of social media, such as Snapchat and Instagram, is significantly associated with vaping, and that celebrity endorsements play a major role in e-cigarette advertising campaigns to form positive perceptions of vaping, especially among young people (5–9). The Centers for Disease Control and Prevention (CDC) declares that e-cigarette use is a risky behavior, and that these toxic and addictive products are harmful, leading to serious health issues, including cancer, impaired brain development, and lung diseases (10). Thus, this study aims to examine college students' perceptions of the risks and benefits of vaping in Saudi Arabia by incorporating the Health Belief Model (HBM). Notably, this study addresses the gap in the literature as limited research has investigated the effects of social media on vaping among college students in Saudi Arabia. The results of this study will help health professionals, educators, and regulators understand the risks posed by e-cigarette advertising campaigns on social media and their effects on college students' behaviors. Moreover, the results will provide practical approaches to address this health issue and regulate e-cigarette use in society.

### Literature review

Existing research on the use of social media shows that individuals are exposed to a large amount of e-cigarette content, which has profound implications on behaviors (8–12). For instance, a study conducted among young people at a large university in the US found that increased social media use, particularly on platforms such as Snapchat and Instagram, was associated with greater exposure to e-cigarette advertising campaigns and higher vaping rates among college students (8). Another longitudinal cohort study of more than 6,000 youth aged 12–17 in the US found that exposure to e-cigarette advertising campaigns on social media not only increased young people's willingness to vape but also lowered their risk perception of e-cigarette use, highlighting the need for regulating online e-cigarette advertising (9). Extensive research shows that exposure to e-cigarette and tobacco content has a profound impact on behavior, as a meta-analysis of 32 studies found a significant association between exposure to e-cigarette or tobacco advertising campaigns on social media and use of these products (11). Another issue is that most individuals who use social media encounter pro-vaping messages. A systematic content analysis of more than 2,900 tweets regarding vaping misinformation revealed that most tweets were pro-vaping and considered vaping to be safe. The study found that among the pro-vaping tweets were news media, which were blamed for spreading misinformation about vaping. Tweets from public health authorities and the news media received the highest engagement, whereas tweets from the tobacco industry received lower engagement (12).

Although the literature suggests that exposure to e-cigarette contents on social media is a significant factor in e-cigarette consumption, other factors like age and sex played crucial roles in vaping consumption. For instance, a study among Middle Easterners found that factors such as younger age and being young and female contributed to early vaping initiation (13). Nonetheless, evidence suggests that studies and literature on vaping in the Middle East have several limitations, including a lack of use of validated measurement tools, and that many more studies are needed to focus on youth and current e-cigarette users (vapers), using validated measurement tools to fill the existing knowledge gaps (14).

### Health belief model (HBM)

HBM is a psychological model widely used in health research to help explain why individuals adopt certain behaviors. The HBM states that beliefs and perceptions influence individuals to embrace certain behaviors (15–17). The HBM consists of six constructs, including perceived benefits, which is considered one of the strongest predictors of adopting certain behaviors. When individuals see that the benefits of a particular behavior outweigh its barriers, they are motivated to adopt that behavior (18). Both perceived susceptibility and perceived seriousness measure individuals' risk perceptions of a particular health behavior (16, 17, 19). Therefore, the integration of HBM to measure risk perceptions and perceived benefits toward vaping is very helpful in understanding the factors that trigger individuals to decide whether to vape or not. In particular, this study focused on key constructs related to e-cigarettes, which are risk perceptions and perceived benefits.

Scholars have extensively investigated factors that motivate individuals to vape. For instance, a study conducted in-depth interviews with 50 minors and adults from a university in the United States and found that the most reported motives of e-cigarette use include smoking cessation, the availability of various flavors, and pleasure and enjoyment (20). Participants also revealed other motives for using e-cigarettes, such as increased focus, relaxation, and for what is called “smoke tricks” (20). Participants perceived vaping as a healthier alternative to regular cigarettes, but also that vaping could carry some potential risks compared to other tobacco products. The study suggests that college students who smoke and vape have lower risk perceptions than those who do not (20). Another study conducted among college students in Taiwan found that non-vapers had significantly higher risk perceptions and lower perceived benefits than people who vape (21). In six European countries, it was also found that current vapers or those who tried vaping but are not currently vaping had significantly lower risk perceptions than those who have never tried vaping (22). Scholars suggest that factors such as perceived benefits and motives play crucial roles in adopting various health behaviors (15, 16, 23). A study investigated motives of e-cigarette use among 562 users during the COVID-19 pandemic in the US and found that motivation is a significant psychological predictor of vaping (24). The study identified three primary motivations that significantly predicted “intensive” vaping among e-cigarette users (24). The first motive was socialization, that is, when vaping is used to replace smoking in a social context when the latter is prohibited. The second motive was health: when individuals feel vaping is less harmful than conventional cigarettes. The study identified a third motive, dependence, that is, when individuals struggle to quit vaping due to their reliance on this behavioral habit (24). Another systematic meta-analysis of 29 studies showed that exposure to tobacco content on social media increases the likelihood of tobacco use and normalization of such behaviors, particularly among young people (25). Another study investigated how social media influenced teens' behavior and attitudes toward vaping (4). They found that most teens believed that vaping is a healthier alternative to conventional smoking, a common idea shared on social media. The study also uncovered that teens perceive vaping as a psychological and emotional tool to reduce anxiety and deal with stress. Teens also believed that vaping is pleasurable, and many had positive perceptions of vaping as a socially appealing behavior to enhance their lifestyle. Many teens admired influencers and celebrities who endorsed vaping behaviors, which shaped teens' perceptions. This created a dilemma in which teens felt that vaping was a great way to belong and be accepted in the community (4). The study added that e-cigarette advertising on social media was crucial in shaping teens' identity, where vaping was portrayed as stylish, fun, and a requirement for social inclusion. Furthermore, curiosity and willingness to experiment with new things were factors that triggered the teens' initial use of e-cigarettes. On the other hand, being aware of the adverse side effects of vaping, such as cancer, was also reported among teens to come from their exposure to health-related content about vaping. These attitudes and behaviors show how social media portrays these products and shapes teens' identities (4).

An earlier study among college students at a large southeastern university in the US also suggested that both e-cigarette users and nonusers perceived vaping as a healthier alternative to regular smoking and a tool to help quit smoking (26). The study incorporated HBM to assess students' perceptions of vaping and found that students lack knowledge about e-cigarettes. Another study among young adults in a country that prohibits the sale and use of e-cigarettes, Singapore, assessed individuals' perceptions toward vaping. The study found that individuals who were exposed to e-cigarette advertising on social media had a positive perception of vaping but did not necessarily vape themselves. The study reported that individuals believed that vaping is more common among young people and that vaping is also common in other countries (27). Another study suggested that HBM was an essential element for increasing college students' awareness of healthy behaviors related to alcohol consumption, resulting in greater adoption of protective behavioral strategies, such as limiting alcohol consumption or changing the manner of drinking (28).

The National Institute of Health and Welfare in Australia stated that more individuals are using vapes, with the majority of vapers in the study trying vaping out of curiosity as the most perceived benefit. The study reported other perceived benefits: 13% of individuals believed that vaping is less harmful than conventional cigarettes, and 12% stated that it tastes much better than cigarettes (29). The American Heart Association stated that many individuals believe that vaping is less harmful than cigarettes and is actually beneficial because it helps to quit smoking; however, that needs more evidence, as vaping is still unsafe (30). Another study examined perceived benefits of vaping among high school students. They found that between 25–33% of students believe that vaping makes individuals comfortable in public events and helps in managing stress. The study also found that 15% believe vaping can make a person more socially desirable and have more friends (31). Another study conducted in Ottawa, Canada, found that enhancing health and aiding in quitting cigarettes were the primary perceived benefits among vapers (32). Therefore, literature shows how psychological factors and exposure to e-cigarette content on social media influence e-cigarette consumption and how individuals view vaping as a healthy alternative to regular smoking. Drawing upon the preceding literature review, this study aimed to:

(1) Estimate the prevalence of smoking and using e-cigarettes among college students;
(2) Assess their levels of perceived benefits, risk perception, overall e-cigarette perception, and exposure to e-cigarette content on social media; and
(3) Examine the associations between perceived benefits, risk perception, overall e-cigarette perception, and vaping behavior; and
(4) Assess perceived harmfulness of e-cigarettes compared to other tobacco products.

We hypothesized that:

(a) Risk perception and exposure to e-cigarette content on social media are associated with regular e-cigarette use among college students,
(b) College students who smoke cigarettes or use e-cigarettes have a lower risk perception of e-cigarettes,
(c) College students who used e-cigarettes in the past 30 days had positive perceptions and greater perceived benefits of e-cigarettes than those who did not, and
(d) There is a correlation between perceived benefits and an overall perception of e-cigarettes.

## Methods

This cross-sectional study utilized non-probability sampling of college students at a large university in Saudi Arabia.

### Instrument

The instrument was based on previously validated and reliable scales related to vaping (10, 24, 27, 33, 34). We assessed content validity by two experts in the field, while construct validity and reliability tests were performed to ensure adequacy. First, a 5-point Likert scale with 7 items (5 = *strongly agree* to 1 = *strongly disagree*) that was based on the CDC's vaping effects on health was adapted to assess risk perceptions. The scale included items assessing risk perception (e.g., “vaping increases the risk of lung cancer”) (10). The reliability of the scale was tested using Cronbach's alpha, and it showed excellent reliability (α = .90). Exploratory factor analysis (EFA) was conducted using principal axis factoring with oblimin rotation to assess construct validity, as it is a robust method in applied research to identify correlated latent constructs in applied research (35, 36). The Kaiser- Meyer- Olkin measure verified the sampling adequacy (KMO = .904), and the Bartlett's test of sphericity was significant, χ^2^(21) = 6,924.62, *p* < .001. The single factor explained 64% of the variance, with all variables loading above .66. A 13-item scale was adapted to measure the perceived benefits of vaping. Example items included “vaping helps me feel better” (24), and the scale demonstrated a high internal consistency reliability (Cronbach's α = .90). Once more, an EFA was carried out using principal axis factoring with oblimin rotation to ensure construct validity. The Kaiser- Meyer- Olkin measure resulted in a sampling adequacy of KMO = .797, and the Bartlett's test of sphericity was significant, χ^2^(78) = 1,076.883, *p* < .001. The three factors explained 57% of the variance, with all variables loading above .40.

Another 8 items assessing overall perceptions of vaping (positive or negative) were also adapted (e.g., “vaping is trendy”), using a 5-point Likert scale (5 = *strongly agree* to 1 = *strongly disagree*) (27). The scale demonstrated satisfactory internal consistency reliability (Cronbach's α = .70). The EFA was once again conducted using principal axis factoring with oblimin rotation. The Kaiser- Meyer- Olkin measure of sampling adequacy was KMO = .771, and the Bartlett's test of sphericity was significant, χ^2^(28) = 603.741, *p* < .001. The two factors accounted for 60% of the variance, with all variables loading above.48.

Furthermore, participants were asked about their smoking and vaping perceptions and behaviors (see Table 1) (10, 24, 27, 33, 34). Items included: Do you smoke cigarettes or use e-cigarettes [yes, no], Do you use e-cigarettes regularly [yes, no], Have you used e-cigarettes in the past 30 days [yes, no]. The participants were also asked about their exposure to e-cigarette advertising on the internet and social media platforms (see Table 2) (10, 24, 27, 33, 34). Items included: In the past 6 months, do you recall seeing any e-cigarette-related content on social media [yes, no, I don't recall], Please indicate in which platform you have seen e-cigarette content [10 platforms such as TikTok and Snapchat]. Other items included: How was the e-cigarette content shared with you [influence/account following, advertisement, I searched it myself, friends, other], How often do you come across any e-cigarette content [daily, at least weekly, monthly, less often than monthly], In the past 6 months, do you recall seeing any e-cigarette content on websites other than social media [yes, no, I don't recall], Which types of websites did you see the e-cigarette content on [news, streaming, blogs, government, etc.]. The study also included demographic questions about factors such as age, sex, and level of education. Questions about the likelihood of using 10 social media platforms, like X (Twitter), Snapchat, TikTok in the past week were used using a 5-point Likert scale (5 = a*lways to 1* = *never*), exposure to vaping content on social media in the past 6 months (*yes, no, I don't recall*), the platforms on which they have seen vaping content, the way vaping content was shared (e.g., *advertisement, influencer/account following, friends*, etc.), and the frequency of encountering vaping content (e.g., d*aily, at least weekly, monthly, etc*.). Participants were also asked about their views on the harmfulness of e-cigarette use (*e.g., less harmful, about the same, and more harmful*) in comparison to other tobacco products, like hookah. Skip logic was applied, in which those who did not use e-cigarettes were intentionally excluded from questions about perceived benefits and overall perceptions of vaping as items in these scales were intended to measure vapers' and smokers' perceptions (*n* = 293).

**Table 1:** Vaping's perceptions and behaviors among college students.

Variables	%
Do you smoke cigarettes or use e-cigarettes? *n* = 1,767	
Yes	16.5
No	83.4
Have you smoked cigarettes in the past 30 days? *n* = 290	
Yes	44.1
No	55.8
Do you use e-cigarettes regularly? *n* = 290	
Yes	66.9
No	33.1
Have you used e-cigarettes in the past 30 days? *n* = 290	
Yes	81.7
No	18.2
How often do you use e-cigarettes? *n* = 289	
More than 4 times a day	62.2
1 to 3 a day	15.5
15.6-7.4,-14.3242ptLess than once a day	22.1
Do you think e-cigarettes make you or others look cool or fit in? *n* = 290	
No	90.3
15.6-7.4,-14.3242ptYes	9.6
Do you think that e-cigarettes are more accepted by nonsmokers? *n* = 1,756	
Definitely	18.3
Probably	41.1
Probably not	14.7
27.6-7.4,-14.3242ptDefinitely not	25.7
Do you think e-cigarettes are allowed in areas where tobacco cigarettes are not allowed (such as in restaurants and movie theaters)? *n* = 1,747	
Yes	14.7
No	67.5
15.6-7.4,-14.3242ptI don't know	17.4
Do you think that e-cigarettes are safe to be used around children? *n* = 1,744	
Yes	3.7
No	96.2

Participants who answered “yes” to do you smoke or vape moved to the next question.

**Table 2:** Social media use and exposure to e-cigarette content.

Variables related to social media use and vaping	Percentages %/M (SD)
1. How often do you use social media every week (e.g.,	
Twitter (X), Snapchat, TikTok, etc.)?	
Daily	90.0%
4-6 days a week	4.3%
1-3 days a week	4.1%
15.6-7.4,-14.3242ptI don't use any type of social media	1.3%
2. When you use social media during the day, please tell us how long you use it.	
More than 3 h	45.1%
Between 1 and 3 h	39.4%
More than 15 min but less than an hour	13.1%
15.6-7.4,-14.3242pt15 min or less	2.3%
3. In the past 6 months, do you recall seeing any e-cigarette-related content on social media platforms?	
Yes	47.4%
No	30.8%
15.6-7.4,-14.3242ptI don't recall	21.6%
4. Please indicate your likelihood of using each social media platform during the past week.	
WhatsApp	4.26 (0.93)
Snapchat	3.75 (1.31)
YouTube	3.57 (1.09)
TikTok	3.37 (1.43)
Instagram	3.15 (1.30)
Telegram	2.98 (1.13)
Twitter (X)	2.90 (1.30)
Pinterest	1.97 (1.13)
LinkedIn	1.85 (1.13)
Facebook	1.16 (0.56)
5. Please indicate on which platform you have seen e-cigarette content. Check all that apply:	
TikTok	33.9%
Twitter (X)	19.5%
Instagram	15.5%
Snapchat	13.1%
YouTube	8.9%
WhatsApp	3.8%
Telegram	1.6%
Pinterest	1.2%
Other	1.1%
Facebook	0.5%
LinkedIn	0.3%
6. How was the e-cigarette content shared with you?	
Influencer/account following	57.5%
Other	16.8%
Advert	15.9%
Friends	6.3%
I searched it myself	3.3%
7. How often do you come across any e-cigarette content?	
Daily	4.4%
At least weekly	30.7%
At least monthly	28.9%
27.6-7.4,-14.3242ptLess often than monthly	35.6%
In the past 6 months, do you recall seeing any e-cigarette-related content on websites other than social media?	
Yes	35.5%
No	41.7%
15.6-7.4,-14.3242ptI don't recall	22.7%
Which types of websites did you see the e-cigarette content on? Check all that apply:	
Entertainment	18.2%
Movie/TV streaming	17.4%
Other	15.7%
News	14.3%
Games	10.1%
Informational website	9.8%
Government	6.0%
Official organization	4.4%
Blog	3.7%

### Procedures

The researchers obtained IRB approval to distribute this study among college students at a large university in Riyadh, Saudi Arabia. Informed consent was obtained from all participants via the online survey prior to the study. To encourage participation, the researchers offered 5 gift cards totaling $134 as incentives, and students who completed the survey became eligible to win one. The university distributed the survey, providing the invitation and the study link via email during the Fall 2,025 semester. A total of 1,897 students participated in the survey, from which a total of 1,680 participants completed it, amounting to an 88.5% completion rate.

### Statistical analysis

Data was analyzed using IBM SPSS (Version 21). Descriptive analyses, such as means, standard deviations, and frequencies were calculated for all variables. Data normality was assessed using the Shapiro-Wilk test, and all variables (adapted scales) violated the normality assumption, *p* < .05. Furthermore, prior to conducting the regression analysis, assumptions were checked, in which linearity and homoscedasticity were satisfied, residuals were normal, and independence of residuals was confirmed (Durbin-Watson = 1.97). The assumptions of binary logistic regression were assessed, in which the dependent variable was binary. The test indicated that all observations were independent and that multicollinearity among predictors was minimal, with the largest condition index of 11.05 and no variance proportions over 0.50. The Box-Tidwell procedure confirmed the linearity of the continuous predictors with respect to the logit, and no influential cases were identified. The sample size was sufficient for three predictors. Thus, the bivariate non-parametric tests, such as Mann-Whitney U, Spearman's rank correlation coefficient, linear regression tests, and binary logistic regression, were performed in this study.

## Results

### Sample characteristics

The study yielded 51% men (*n* = 975) and 49% women (*n* = 922). Regarding the participants' educational background, 67.4% of students indicated that their highest level of education was high school (*n* = 1,271), 22.2% had a bachelor's degree (*n* = 419), 8.5% had a master's degree (*n* = 160), and almost 2% had doctoral degrees (*n* = 37). Around 79% of students were between 18 and 24 years old (*n* = 1,482), 14% between 25 and 34 years old (*n* = 267), 6% between 35 and 44 years old (*n* = 113), and less than 2% were above 45 years old (*n* = 22).

The descriptive analyses indicated that 83% of college students do not smoke cigarettes or use vapes (*n* = 1,474), and around 17% indicated they smoke or use vapes (*n* = 293). Among those who indicated that they smoke cigarettes or use e-cigarettes more than 77% stated that they do not smoke cigarettes regularly (*n* = 225) compared to 23% who said they smoke cigarettes regularly (*n* = 66). Table 1 shows vaping-related perceptions and behaviors.

More than 90% of college students reported using social media daily (*n* = 1,683), and 45% reported using it more than 3 h a day (*n* = 825). WhatsApp was the most commonly used platform (M = 4.26, SD = 0.92), and Facebook was used the least (M = 1.16, SD = 0.56). Respondents reported that TikTok was the platform with the greatest amount of vaping content (M = 4.26, SD = 0.93). Other findings related to exposure to vaping content are shown in Table 2.

Overall, students indicated high risk perception of e-cigarette use (M = 4.08, SD = .88). Most students strongly agreed that vaping can increase the risk of lung cancer (M = 4.47, SD = .81), others agreed that vaping can be harmful to brain development (M = 4.14, SD = .96), and others agreed that vaping can make them irritable (M = 4.05, SD = 1.13).

Moreover, the students who vape had positive perceptions of vaping (M = 4.06, SD = 0.59), such as vaping is more common among young people than older people (M = 4.45, SD = 0.87) and vaping is trendy (M = 4.41, SD = 0.84). Also, vapers held neutral perceptions with the statement that vaping is relaxing and enjoyable (M = 3.02, SD = 1.27).

Table 3 shows students' perceptions of the harmfulness of vaping, compared to other tobacco products.

**Table 3:** Comparison of e-cigarettes with other tobacco products.

Products	Less harmful %	*n*	About the same %	*n*	More harmful %	*n*
E-cigarettes	13.3	230	41.8	720	44.7	769
Chewing tobacco	26.0	448	46.4	799	27.4	472
Shisha/Hookah	7.4	128	36.5	628	56.0	963
Snus	19.9	342	58.6	1008	21.4	369
Dissolvable nicotine products	20.7	356	46.7	803	32.5	560

*N* = 1.719.

The overall perceived benefits of e-cigarette use were moderate (M = 3.20, SD = .70). The highest perceived benefits among students were that “vaping comes in flavors that I like” (M = 3.76, SD = 1.14), followed by “vaping is more acceptable to nonsmokers” (M = 3.69, SD = 1.11), and “vaping might be less harmful to others around me than cigarettes” (M = 3.68, SD = 1.27).

A binary logistic regression was conducted to examine whether risk perception and exposure to vaping content on social media were associated with regular vaping. The overall model was statistically significant, χ^2^(2) = 15.17, *p* = .001, and the model explained 6.5% of the variance in vaping (Cox & Snell R^2^) and 9.2% according to Nagelkerke R^2^ (see Table 4).

**Table 4:** Association of exposure to e-cigarette content, risk perception, and e-cigarette use.

Predictor	B	SE	Wald	df	*p*	OR Exp(B)	95% CI for OR	
Risk perception	−0.077	0.026	9.173	1	.002	0.93	0.88-	0.97
E-cigarette content exposure	−0.500	0.351	2.033	1	.154	0.61	0.31-	1.21
Constant	2.772	0.579	22.958	1	.000	16.00	—	—

The outcome variables in the logistic regression were coded as no = 0 and 1 = yes.

An increase in risk perception was associated with lower odds of vaping (OR = 0.93, 95% CI [0.88, 0.97], *p* = .002). However, exposure to vaping content on social media was not a statistically significant predictor of vaping [OR = 0.61, 95% CI (0.31, 1.21), *p* = .154].

The bivariate Mann-Whitney analysis also showed significant differences in risk perceptions between students who smoke cigarettes or vape and those who do not (U = 48,961.0, *p* < .001). College students who smoke cigarettes or vape had lower risk perceptions (Mdn = 3.0) than those who do not (Mdn = 4.42). The Mann-Whitney test also indicated significant differences in risk perceptions between regular vapers and non-vapers (U = 5,594.0, *p* < .001). Regular vapers had a lower risk perception (Mdn = 2.85) than those who do not regularly vape (Mdn = 3.28).

Moreover, the bivariate Mann-Whitney test revealed a significant difference in perceived benefits between students who had vaped in the past 30 days and those who had not (U = 1,987.0), *p* < .001. Students who vaped in the past 30 days had higher perceived benefits (Mdn = 3.30) than those who had not vaped in the past 30 days (Mdn = 2.61). The Mann-Whitney test (U = 3,176.0, *p* < .01) also indicated that students who vaped in the past 30 days had more positive perceptions toward vaping (Mdn = 4.25) than those who did not vape (Mdn = 4.0).

The Spearman's rank correlation coefficient showed that perceived benefits positively correlated with the overall perceptions of vaping (*p* < .001; r = .332). Furthermore, a linear regression analysis found that perceived benefits predicted students' overall perception of vaping, F (1, 242) = 59.755, *p* < .001, R^2^ = .198. Higher perceived benefits were associated with more positive overall perceptions of vaping (B = 0.228, β = .445, t = 7.730, *p* < .001), with a 95% confidence interval ranging from 0.170 to 0.286.

## Discussion

This study elucidates college students' perceptions of vaping, their use of social media platforms, and their exposure to vaping-related content on various platforms. Although the study was conducted at one of the largest universities located in Riyadh, the capital city of Saudi Arabia, with a diverse student body, the majority of college students (83%) reported never having smoked cigarettes or vaped. This is similar to a study conducted across three universities in the United Arab Emirates, which found that more than 81% of college students do not vape or smoke (37). It is also comparable with the findings of a meta-analysis of 29 studies concerning nicotine consumption in Saudi Arabia, which found that only 17% of college students consume tobacco products (38). Such promising findings can be attributed to the level of awareness college students possess of the health risks posed by smoking and vaping. Another contributing factor may be the national implementation of regulations, taxes, and policies that discourage young people from vaping. A study could provide some insights into how regulations could influence individuals' behaviors (39). It was found that there was a sharp decline in the number of college students who smoke cigarettes and vape in the state of Texas between 2015 and 2024, except for a spike in e-cigarette use between 2017 and 2019 due to the popularity of pod vapes and the brand JUUL. The study highlighted that FDA regulations and tobacco policies contributed to a decline in the vape and smoking market in Texas between 2019 and 2024, and emphasized the importance of monitoring the tobacco industry to develop effective strategies to counter the promotion of unhealthy products (39).

Nevertheless, we found that almost half of college students have been exposed to vaping-related content on social media, such as TikTok, X (Twitter), Instagram, and Snapchat, or other media, such as entertainment websites, movies, and streaming services. More than half of those college students were exposed to vaping content through influencer or followed accounts, and less than a quarter were exposed via advertising. Furthermore, our study concludes that college students possess high risk perceptions regarding vaping, with the majority of college students believing that vaping can cause serious health issues (including cancer and impaired brain development) and make them more irritable. This is also similar to a study that found that the risk perceptions of vaping in comparison to conventional cigarettes have been increasing among adults in the United States, as both products pose serious health risks (40). However, former and current users held less risk perceptions than those who had never smoked or vaped (40). Many studies confirm that college students who vape have lower risk perceptions than those who do not (21, 22, 41, 42), which aligns with our findings that college students who smoke or vape have lower risk perceptions of vaping compared to those who do not.

Our study has also suggested that more than 90% of college students use social media daily, and almost half of college students have seen vaping-related content on social media; however, the majority do not vape or smoke. Unlike previous studies focusing on teens and adolescents (4, 5, 9, 43), our study did not find significant associations between exposure to vaping content on social media and regular vaping among college students. In particular, the regression model indicated that exposure to vaping content on social media was not associated with regular vaping, whereas with modest explanatory power, higher risk perception was associated with lower e-cigarette use. The absence of a significant association between exposure to e-cigarette content on social media and regular vaping as well as the modest explanatory power of the association between risk perception and e-cigarette use in this study could be attributed to other factors influencing human behaviors, such as social norms, psychological characteristics, and peer influence and that exposure to social media content can only be one indirect factor influencing behaviors (44–46). Also, the way exposure was measured in this study could be another possibility. Particularly, simply asking whether students had seen vaping-related content may not fully capture the strength or nature of that exposure. This is important, as the influence of social media often depends on how frequently the content is seen, who shares it, how it is presented, and how persuasive or appealing the message is (47). Furthermore, non-vaper college students showed they have high risk perceptions of vaping, which may indicate that they possess higher health literacy. This is comparable to a study that found that female and non-vaper college students carry higher risk perceptions than students who vape. The study found that college students who use vapes and study in a field other than health and medicine have lower risk perceptions toward vaping than college students majoring in health (41). Such findings are encouraging, as increasing health education and awareness campaigns in universities in Saudi Arabia may deter non-users from starting to vape, as vaping can serve as a starting point to other tobacco products (41). Tailored messages that address risk perception are indispensable for reducing peer pressure and feelings of not fitting in among non-vapers in campus communities. This is crucial, as a recent study found that students form friendships based on similarities in vaping and smoking behaviors and that peer influence plays a vital role in vaping initiation and persistence among students (48). Thus, tailored health campaigns that understand the psychology of college students can be effective, especially at larger schools that have higher rates of smoking or vaping. Perhaps INTENT prevention programs, in which teachers delivered engaging lessons to students, resulting in increased students' knowledge about the harms of vaping and reduction of its use in schools in Great Britain, could be implemented across schools in Saudi Arabia to increase students' risk perception and slow the widespread use of vaping (49). Similar programs are proven effective in not only raising awareness of smoking and vaping risks, but also in addressing the risks of substance and drug use among students. For instance, a program called “Ho‘ouna Pono” launched in Hawaii schools, integrated a culturally grounded school-based drug prevention curriculum that allows teachers to use video lessons, interactive activities, and real-life scenarios to prevent drug use among youth (50). Such strategies can be adopted across schools and universities in Saudi Arabia to reduce the use of e-cigarettes.

Our findings also show that college students who had vaped in the past 30 days held higher perceived benefits than those who had not. Examples include “vaping comes in flavors I like”, “vaping is more acceptable to nonsmokers”, and “vaping is less harmful than conventional cigarettes,” which are some of the main perceived benefits reported among college students, which are aligned with the literature. For instance, a study found that non-vapers, compared to those who vaped in the past 30 days, believed that vaping is more harmful to health (31). Another research also found that college students who vaped had high perceived benefits, including that vaping is less harmful to health, less addictive, and a great alternative to conventional cigarettes (51). The findings are also comparable to a study, which found that vapers had higher perceived benefits and lower risk perceptions than non-vapers (21). Our study also found that the majority of college students who vape do not feel that vaping makes them “cool” or helps them to fit in.

Furthermore, our findings suggest a significant positive association between perceived benefits and the overall perceptions of vaping. Those students who perceive vaping to have some benefits hold more positive perceptions toward vaping. This is comparable to studies that found that higher perceived benefits are associated with a positive intention to vape (31, 51, 52).

### Limitations

One key limitation is that the study relied on a single tool—a self-administered survey—which may overlook important nuances and is vulnerable to typical self-report bias. Other unmeasured factors, such as stress, peer influence, and sensation seeking, could have also influenced vaping behavior and risk perceptions. Another limitation is the use of purposive sampling, which limits the generalizability of the findings of this study. Future research could benefit from a mixed-methods approach that provides a clearer, more grounded picture of how students understand the risks and benefits of vaping, and how their social environment and personal experiences shape those views.

## Conclusion

This study examined college students' perceptions of vaping in relation to their social media use and exposure to vaping-related content. Interestingly, the study did not find a significant association between exposure to vaping- related content on social media and vaping behavior. Rather, students' decisions about vaping may be shaped by various daily life factors, such as peer pressure, the surrounding social environment, personal beliefs, stress levels, and the kind of vaping-related messages they frequently encounter online. The findings show that students who use e-cigarettes have lower risk perceptions and view vaping as less risky than students who do not. The study also suggests that perceived benefits matter; some students may be attracted to vaping because they see it as enjoyable, socially acceptable, or less harmful than smoking. Taken together, these findings suggest that universities and health professionals need to address vaping in a more practical and realistic way—not only by explaining its health risks, but also by challenging the beliefs, social pressures, and positive images that make vaping seem normal or harmless to some students.

## Data Availability

The datasets generated and/or analyzed during the current study are available from the corresponding author upon reasonable request.
